# Evaluating the Antimicrobial Properties of Different Dental Cleaning Tablets Against *S. aureus* Grown on Two Denture Base Materials

**DOI:** 10.1002/cre2.70189

**Published:** 2025-07-31

**Authors:** Sindi Vishaj, Alissa Villhauer, Mandi Morris, Donald M. Belles, Daher Antonio Queiroz, Maria D. Gonzalez, Ransome van der Hoeven

**Affiliations:** ^1^ Department of Restorative Dentistry and Prosthodontics University of Texas Health Science Center at Houston School of Dentistry Houston Texas USA; ^2^ Iowa Institute for Oral Health Research University of Iowa College of Dentistry Iowa City Iowa USA; ^3^ Department of Diagnostic and Biomedical Sciences Houston Texas USA; ^4^ Department of Periodontics University of Iowa College of Dentistry Iowa City Iowa USA

**Keywords:** dentures, Efferdent, Polident, *Staphylococcus aureus*, Val‐clean

## Abstract

**Objectives:**

This study aimed to evaluate the antimicrobial efficacy of three commercially available denture cleaning tablets Efferdent, Polident, and Val‐clean against *Staphylococcus aureus* biofilms formed on two denture base materials: milled polymethyl methacrylate (PMMA) and 3D printed denture resin.

**Material and Methods:**

Eighty samples were fabricated, with 40 specimens per denture base material: CAD/CAM milled PMMA (Lucitone Digital Fit LT; Dentsply Sirona, Charlotte, NC, USA) and 3D printed resin (Denture Base Resin; Formlabs Inc., Somerville, MA, USA). Each group was divided into four subgroups (*n* = 10), Efferdent, Polident, Val‐clean, and deionized water (control). Surface roughness was measured using a digital profilometer (Digiprofilo I; Digiwork Instruments, Concord, ON, Canada). Samples were exposed to *S. aureus* in tryptic soy broth (TSB) and subsequently treated with the cleaning solutions. Colony‐forming units (CFUs) were quantified using serial dilution plating. To distinguish bactericidal from bacteriostatic effects, normalized *S. aureus* cultures were incubated with the cleaning agents for 16 h, followed by optical density measurements, LIVE/DEAD staining (Invitrogen, Carlsbad, California), and TSB agar plating. Statistical analysis was performed using one‐way ANOVA and Dunnett's multiple comparisons test, with significance set at *p* < 0.05.

**Results:**

A significant difference in surface roughness was observed between the 3D printed and milled samples before treatment (*p* < 0.0001). Significantly fewer CFUs were observed in all cleaning solution groups compared to the control (*p* < 0.05). All denture cleaning solutions demonstrated bactericidal activity against *S. aureus*, as indicated by the absence of CFU growth on agar plates and the lack of viable cells in LIVE/DEAD staining posttreatment. Both denture materials demonstrated comparable microbial adhesion and response to the cleaning agents.

**Conclusions:**

Efferdent, Polident, and Val‐clean denture tablets are effective bactericidal agents against *S. aureus* in vitro, regardless of the denture base material used. These findings support their use in daily denture hygiene routines, particularly in reducing bacterial colonization on removable prostheses.

## 1 Introduction

Complete edentulism is defined as the loss of all permanent teeth (The glossary of prosthodontic terms 2005). In the United States, approximately 26% of the individuals aged 65 and 74 are completely edentulous (Al‐Rafee 2020). The need for dentures has been estimated to be 37.9 million adults in the US alone (Douglass et al. 2002). Complete dentures, like natural dentition, can accumulate plaque, stain, and calculus. Denture plaque consists of a complex mixture of bacteria, fungi, and other microorganisms. Inadequate removal of biofilm from dentures is linked to higher occurrence of localized denture stomatitis, as well as more severe systemic diseases (Neppelenbroek 2015). Denture stomatitis frequently affects denture wearers, manifesting as erythema beneath the denture (Hannah et al. 2017). Its causes are multifactorial, involving both local and systemic factors (Gendreau and Loewy 2011). Although *Candida albicans* has been most correlated with denture stomatitis, the emergence of methicillin‐resistant *Staphylococcus aureus* has become a major issue in hospitalized and elderly patients (Gad and Fouda 2020; Honma et al. 1994). A recent study found that Staphylococcal strains isolated from denture wearers triggered greater activation of monocytes but were less susceptible to phagocytosis (Garbacz et al. 2019). The reduced phagocytic efficiency against biofilm‐forming *S. aureus* in denture wearers suggests colonization by strains that are more resistant to immune clearance compared to those found in non‐wearers. This resistance may contribute to increased colonization and infection rates, particularly among vulnerable populations such as the elderly.

In 2009, the American College of Prosthodontists established a task force dedicated to crafting evidence‐based guidelines for the care and maintenance of complete dentures. Some of these guidelines include reducing levels of biofilm by daily soaking and brushing of complete dentures with an effective, nonabrasive denture cleaner (Felton et al. 2011). The most common methods for removing denture plaque conducted by patients have been divided into two groups: mechanical and chemical methods (de Souza et al. 2009; Mylonas et al. 2022). For the mechanical cleaning of the removable prostheses, brushes and ultrasonic devices have been reported (Schmutzler et al. 2021). Since 1924, brushing with warm water has been the most recommended denture cleaning method. A significant disadvantage of mechanical brushing can be potentially harmful and can result in wear of dentured acrylic resin (Melo et al. 2021). The chemical method involves using immersion‐type chemicals, which can be divided into two major groups: denture cleansers and disinfectants (Nikawa et al. 1999). These effervescent tablets utilize a wide range of active agents, but the most common ones are hypochlorite agents, peroxides, enzymes, acids, and oral mouth rinses. Each cleanser operates through a distinct mode of action and varies in efficacy for removing denture biofilm. An ideal denture cleanser should possess several characteristics such as nontoxic, short‐acting effect, user‐friendly, acceptable taste or tasteless, and cost‐effective. The chemical composition of the tablets or solutions should not alter or degrade the surface of acrylic denture bases or prosthetic teeth. Although tested denture cleaning methods are successful in reducing the biomass, none of the reviewed in vivo trials showed bactericidal effects (Felton et al. 2011).

Some examples of the most common denture cleansers include the peroxide, monopersulfate, and oxidizing‐type cleansers. Hypochlorite agents are effective for disinfecting dentures, and they dissolve organic matter on which tartar is formed (Mylonas et al. 2022). This type of denture cleaner has demonstrated a greater ability to remove stains from removable prostheses. A drawback of these agents is their ability to corrode metals. Polident and Efferdent tablets belong to the monopersulfate group, which replaces perborates or percarbonates. Newer formulations are less alkaline and tend to be in the neutral range. These oxidizing agents are incorporated to aid in stain removal in addition to their antibacterial effects. According to the manufacturer's information, residual plaque left after use of peroxide cleansers contains low levels of viable microorganisms, and a reduction of 99% in plaque organisms after 15 min of soaking was reported. The research data supporting these claims have not been published (Denture cleansers. Council on Dental Materials, Instruments, and Equipment 1983).

Materials used in the fabrication of dentures can impact the binding and colonization of microorganisms due to changes in surface properties (Nassary Zadeh et al. 2020). Polymethyl methacrylate (PMMA) is a widely used material in dentistry, consisting of fine powder particles and monomer liquid. Despite their advantages in cost, biocompatibility, and quick fabrication, PMMAs have various drawbacks including polymerization shrinkage, thermal changes, and surface porosity. Dental prostheses made from PMMA are prone to bacterial colonization due to their rougher surfaces and unpolished surface of the intaglio of the restorations (Nassary Zadeh et al. 2020).

Computer‐based design and manufacturing typically fall into two categories: subtractive or additive manufacturing. These two categories have been successfully incorporated to create dentures. In the 1980s, three‐dimensional (3D) printing was first introduced, and significant advancements have occurred throughout the following decades. 3D printing is easily accessible, user‐friendly, and dependable. The main 3D printers in dentistry are classified based on their technology: stereolithography (SLA), selective laser sintering (SLS), or digital light processing (DLP) (Rayyan et al. 2015). SLA employs UV light to solidify liquid photopolymer layers, while DLP uses a digital projector for rapid curing of entire layers. Both SLA and DLP offer precision and accuracy, with SLA printers being faster for small to medium‐sized objects. Another additive manufacturing technology is fused deposition modeling, which involves melting thermoplastic filaments and extruding them through a heated nozzle onto a building platform. Fused deposition modeling has limitations due to material constraints (Dizon et al. 2018; Schweiger et al. 2021).

In the subtractive technology of CAD/CAM, denture base materials are milled from prefabricated PMMA blocks, ensuring high accuracy and material properties. CAD/CAM systems enable precise shaping of materials, reducing bacterial contamination risk and enhancing fit (Zafar 2020). CAD/CAM technology has drawbacks, including errors in milling bur diameters, potentially causing inaccuracies and material waste. Milled complete dentures may lack a natural appearance due to monochromatic bases. Milling yields homogenous structures with minimal post‐processing adjustments. Compared to conventionally fabricated complete dentures, CAD/CAM dentures demonstrate either a similar or superior fit of the intaglio surface. Printer parameters like printing orientation, layer thickness, laser intensity, and speed affect the properties of printed products. These parameters can be modified to achieve the desired outcome.

Unlike other studies where *C. albicans* has been the microorganism of choice, the aim of this investigation was to evaluate the antimicrobial properties of commonly used denture cleaning tablets against *S. aureus* grown on two denture base materials. Research comparing these common denture cleaning tablets against *S. aureus* is limited. Findings of this study may aid clinicians in recommending the appropriate denture cleaning material when providing hygiene instructions to patients after delivery of their dental prostheses. The null hypothesis for this study states that there is no significant difference between different dental cleaning solutions in the removal of *S. aureus* compared to sterilized deionized water (DW) in two different denture base materials.

## 2 Materials and Methods

### 2.1 Materials Used in This Study

This in vitro experimental study aimed to evaluate the bactericidal efficacy of three commercial denture cleaning tablets (Polident, Efferdent, Val‐clean) against *S. aureus* on two types of denture base materials: 3D printed resin and milled PMMA. A total of 80 samples were fabricated, and 40 specimens were tested per material group (Table 1). Each material group was further divided into four subgroups (*n* = 10) based on the treatment solution used: Group 1: Control (Sterile DW), Group 2: Polident (Haleon, Brentford, UK), Group 3: Efferdent (Prestige Consumer Healthcare Inc., Tarrytown, NY, USA), and Group 4: Val‐clean (Valplast International Corp., Nesconset, NY, USA) (Table 2). The sample size (*n* = 10 per subgroup) was determined based on a pilot study and was considered sufficient to detect statistically significant differences using one‐way ANOVA and post hoc comparisons with an alpha level of 0.05.

**Table 1:** Description of materials used in this study.

Polymerization method	Material	Type	Manufacturer
3‐D Printing	Formlabs Denture base resin	Liquid resin	Formlabs Inc., Somerville, MA, USA
CAD/CAM PMMA disc milling	Lucitone Digital Fit LT,	Prepolymerized polymethyl methacrylate (PMMA)	Dentsply Sirona

**Table 2:** Number of samples used in each subgroup.

Groups	Control group: deionized water	Efferdent	Polident	Val‐clean
3D Printed	10 samples	10 samples	10 samples	10 samples
Milled PMMA	10 samples	10 samples	10 samples	10 samples

### 2.2 Preparation of Denture Materials

**Figure 1:**
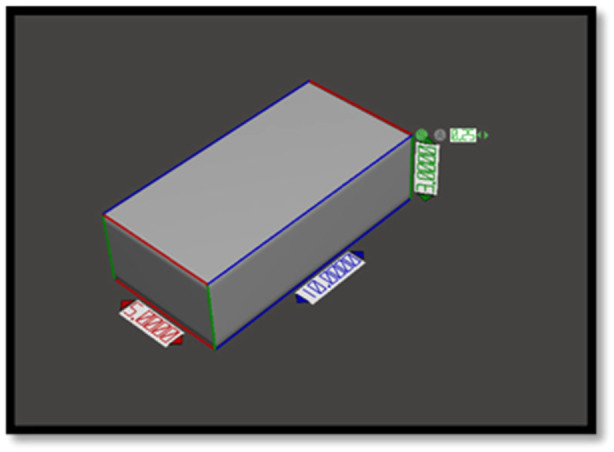
STL file of 3D designed sample in (Meshmixer; Autodesk Inc., San Rafael, CA) software.

The 3D printed denture base resin OP (Formlabs Inc.) was first prepared as follows. A STL (Standard Tessellation Language) file of the samples was designed using 3D modeling software (Meshmixer; Autodesk Inc., San Rafael, CA) (Figure 1). The samples were printed in 10 × 5 × 3 mm dimensions and at 50 μm layers line resolution using an in‐office stereolithography 3D printer (Form 3B+; Formlabs, Somerville, MA) oriented at 0 degrees on a brand‐new stainless‐steel build platform. Samples were printed from the same 3D printer and platform randomly assigned to a test subgroup. Printed samples were washed in Formwash in 99% alcohol for 10 min and dried (Denture Base Resin, n.d.). The samples were removed from the build platform by wedging with a sample removal tool. The supports from the printed samples were cut off, and the samples were light‐cured using the following steps. A glass container was filled with glycerin which was preheated to 800°C in the Form Cure. The samples were fully submerged in the glycerin container and were cured initially for 30 min. Once the initial cure was completed, the samples were flipped to the opposite side and cured for an additional 30 min. The milled PMMA samples were prepared from a pre‐polymerized disc (Lucitone Digital FitTM LT, Dentsply Sirona) by a five‐axis mill using the same STL file. The same orientation was maintained for all samples inside the pre‐polymerized puck.

### 2.3 Roughening Protocol

The same roughening force and surfaces were applied for each group. For the 3D printed samples, we used acrylic burs to remove supports and smooth touchpoint areas. A sample holder was used to position each sample identically. Only the surface where supports were removed was roughened using 220‐grit sandpaper under 2 kg of consistent pressure to simulate the rough intaglio surface that contacts oral tissues. The same sequences were utilized for the milled PMMA samples, starting with acrylic burs to remove the supports for two opposing sides of the samples. The same sample holder was used to position each sample. Two sides of the milled PMMA samples were finished using sandpaper with fine grit (220) and 2 kg force. The rationale for treating two surfaces in milled PMMA versus one surface in 3D printed samples was to account for the difference in support structures and finishing needs inherent to each manufacturing process. Milling produces uniform surfaces, requiring more area to be intentionally roughened to match the microbial adhesion potential of the support‐affected 3D printed surfaces. All 3D printed and milled PMMA samples were placed in a bag of distilled water for 10 min in an ultrasonic bath (Cole‐Parmer; Fischer Scientific, USA).

### 2.4 Surface Roughness Measurements

To measure surface roughness (Ra), a pre‐calibrated digital profilometer (Digiprofilo I; Digiwork Instruments, Concord, ON) was used. The instrument consists of a general‐purpose piezoelectric probe (SFP‐2001), and a Ra measurement range (0.03–6.3 μm/1 μm), cut‐off wavelength 0.8 mm, and traverse length of 3.00 mm. The sample holder was used to stabilize each sample. For the 3D printed group, the Ra values were recorded only for the roughened surface. For the milled PMMA group, the Ra values were recorded for two surfaces. The readings were performed at three points for each specimen, and the mean of the three values was calculated and recorded as the Ra. The digital profilometer was calibrated between samples to ensure consistent accuracy. All other surfaces of the 3D printed and milled PMMA samples were also measured, but they exhibited similar Ra values, as the manufacturing processes produced comparably roughened surfaces.

### 2.5 Preparation of Denture Cleaning Solutions

All the denture cleaning solutions were prepared following the manufacturer's recommendations. If the manufacturer did not have any specifications about the volume of water used to dissolve the tablets, the solutions were prepared based on clinical guidelines to closely represent patients' protocols. A denture cup was used to measure the volume of water that patients use. This volume was determined to be 100 mL. All the calculations for Efferdent and Polident were based on a final volume of 100 mL. For Polident, one tablet weighed 2.746 g. The tablet was crushed into a powder form, and a balance was used to measure 0.275 g. The powder was dissolved in 10 mL of DW by vortexing for 1 min. The same protocol was followed for Efferdent tablets. One tablet of Efferdent weighed 2.067 g. The tablet was crushed, and 0.207 g of Efferdent powder was mixed with 10 mL of DW. On the other hand, Val‐clean solution was formulated using the instructions provided in the kit. 0.017 g of Val‐clean powder was added to 10 mL of DW, and the solution was vortexed for 1 min. All solutions were freshly prepared for each replicate of the experiment.

### 2.6 Microbial Adhesion Assay

Cultures of *S. aureus* NCTC 8325 were grown overnight in Tryptic Soy Broth (TSB) at 37°C on an orbital shaker (Barnstead MaxQ 4000 Digital Orbital Incubator Shaker, Marshall Scientific, Hampton, NH) at 200 rpm. The cultures were normalized to an Optical Density (OD_600nm_) = 0.1 using an Eppendorf BioPhotometer (Eppendorf AG, Hamburg, Germany). All 3D printed samples and milled PMMA samples were sterilized on both sides using ultra‐violet light for a total duration of 30 min. Using sterile forceps, the samples were placed into tubes containing normalized cultures of *S. aureus*. The samples were incubated at 37°C on an orbital shaker for 24 h. Subsequently the tubes were removed from the shake and were incubated under static conditions at 37°C for an additional 48 h. Using sterile forceps, the inoculated samples were transferred to their corresponding tubes with 500 μL of each denture cleaning solution and sterile DW. The samples were incubated in the respective cleaning solution at room temperature for 8 h. Thereafter, the samples were washed in phosphate‐buffered saline (PBS) and transferred to tubes containing 500 μL PBS and subjected to sonication in an ultrasonic bath for 30 s three times. The samples were vortexed for a minute, and 10‐fold serial dilutions of each sample were prepared and plated on TSB agar. The plates were incubated at 37°C overnight, and the number of CFUs was counted for each dilution. All experiments were performed in three independent sessions.

### 2.7 Growth Assay

Isolated colonies of *S. aureus* were inoculated into 2 mL of TSB media using a sterile applicator. Cultures were grown on an orbital shaker overnight at 37°C. Subsequently, cultures were normalized to an OD_600nm_ of 0.05. 100 μL normalized culture was added to each well in a 96‐well microtiter plate. 2× concentrations of each denture cleaning solution were prepared as described previously and added to the wells containing the normalized cultures. Sterile DW was used as controls. The plate was incubated at 37°C while shaking at 200 rpms for 16 h. The OD was measured using a Tecan plate reader after 16 h of growth. Subsequently, 10 μL of the contents of each well was plated onto TSB agar, incubated overnight at 37°C, and observed for growth on the next day. The experiment was replicated three times. Additionally, cultures were centrifuged at 13,000 rpm for 1 min, and the resulting cell pellet was washed with PBS to remove any residual media. The cells were then stained with SYTO9 and propidium iodide (1:1000 dilution) for 15 min in the dark. Stained cells were visualized using an Olympus BX63 fluorescence microscope.

### 2.8 Statistical Analysis

Statistical analysis for all experiments were performed using GraphPad Prism version 10.0 (GraphPad Software, San Diego, CA). One‐way ANOVA was conducted to determine statistical significance among all samples and a Dunnett's multiple comparisons test was used to determine statistical significance between samples. A *p* < 0.05 was considered significant in all experiments.

## 3 Results

**Figure 2:**
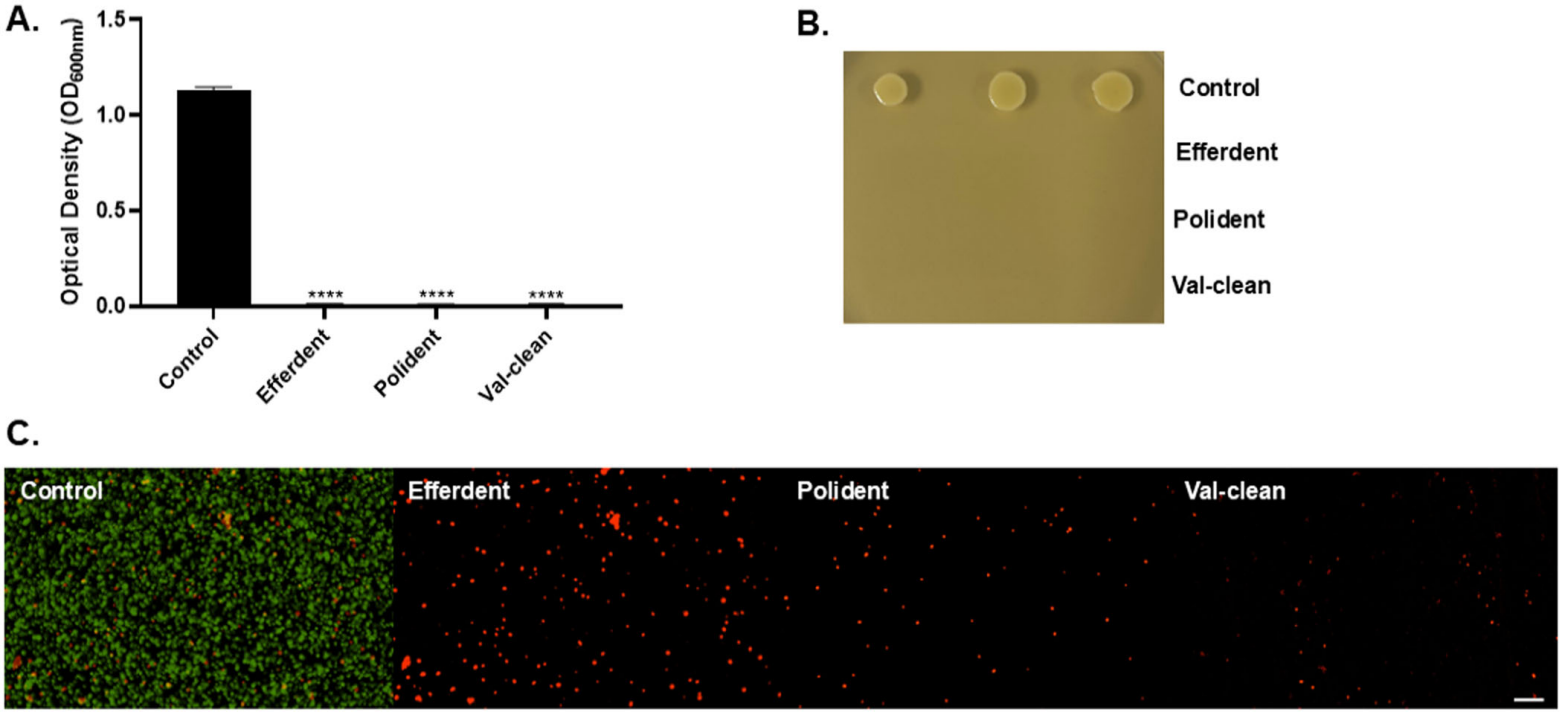
(A) Optical density (OD = 600 nm) of *S. aureus* grown in the presence of Efferdent (*p* < 0.0001), Polident (*p* < 0.0001), and Val‐clean(*p* < 0.0001) solutions after 16 h of incubation relative to the control. (B) A Representative image of 16 h grown cultures in the presence of Efferdent, Polident and Val‐clean and control spotted onto TSB plates. (C) Representative fluorescence microscopy images of LIVE/DEAD‐stained *S. aureus* cultures after 16 h of incubation with Efferdent, Polident, Val‐Clean, and the control. Green fluorescence indicates live cells, while red fluorescence denotes dead cells.

A significant difference in surface roughness was observed between the 3D printed and milled samples before treatment (Table 3, *p * < 0.0001). Additionally, all denture cleaning solutions tested resulted in a statistically significant reduction in microbial adhesion compared to the control group for both materials (Table 4). Among the 3D printed samples, the reduction was highly significant (*p* < 0.0001), while the milled PMMA samples also showed a significant decrease in microbial adhesion (*p* = 0.001) relative to the control. Each material group was analyzed relative to its respective control. The growth assay further supported these findings, with optical density measurements after 16 h of incubation indicating significantly lower microbial growth in all denture cleaning solution groups compared to the control (Figure 2A). Additionally, LIVE/DEAD staining revealed substantial bacterial death when treated with denture cleaning solutions relative to the control (Figure 2B). No visible growth was observed on TSB agar plates for any samples treated with denture cleaning solutions, in contrast to those exposed to sterile DW (Figure 2C).

**Table 3:** Surface roughness (*R*_a_) of the groups (*n* = 40) after fabrication.

Group	Mean	Standard deviation
3D printed	0.64	0.07
PMMA	0.54	0.03

**Table 4:** Number of *S. aureus* CFUs/mL adhered to 3D printed and Milled PMMA samples in the presence of Efferdent, Polident, and Val‐clean.

Sample	Control	Efferdent	Polident	Val‐clean
3D printed	1.59 × 10^5^ ± 7.4 × 10^4^	0	0	0
PMMA	1.64 × 10^5^ ± 6.25 × 10^4^	0	0	0

## 4 Discussion

The null hypothesis for this in vitro study stated that there would be no significant difference in the removal of *S. aureus* between different denture cleaning solutions and sterile DW across two denture base materials. Based on the findings, the null hypothesis was rejected, as all tested cleaning agents (Polident, Efferdent, and Val‐clean) significantly reduced bacterial adhesion and exhibited bactericidal effects against *S. aureus* compared to the control.

This study evaluated the antimicrobial efficacy of three commonly available denture cleansers on CAD/CAM milled PMMA and 3D printed denture base resins, which are both increasingly utilized in modern prosthodontics. Results demonstrated that all three denture cleaning solutions significantly reduced the number of *S. aureus* CFUs on both types of denture base materials. Val‐clean showed slightly superior antimicrobial performance among the three, as seen by the LIVE/DEAD staining, although no growth was detected on agar plates for any cleaning solution, indicating complete bactericidal activity. Val‐clean is specifically formulated for Valplast flexible dentures. Unlike PMMA, Valplast is a thermoplastic polyamide made of nylon resin. Our data supports the ability of Val‐clean formulated for Valplast flexible dentures can eradicate *S. aureus* grown on both denture base materials, as seen with Polident and Efferdent.

Surface roughness is a critical factor influencing microbial adhesion. The Ra values were intentionally kept within clinically relevant unpolished ranges to simulate the intaglio surface of dentures, which is typically not polished. 3D printed samples exhibited higher surface roughness than milled PMMA, yet both materials supported substantial microbial adhesion in the absence of cleaning solutions, highlighting the need for effective hygiene protocols. A recent study reported that new‐generation denture base materials fabricated through additive, subtractive, and conventional methods initially exhibited unacceptable surface roughness levels (> 0.2 μm) before polishing (Çakmak et al. 2024). Polishing significantly reduced roughness to clinically acceptable levels (< 0.2 μm). Notably, denture cleansers did not significantly alter surface roughness, and the materials maintained acceptable roughness after cleansing. However, another study reported that Corega, chlorhexidine, and hydrogen peroxide significantly impacted the surface properties of heat‐polymerized denture base materials (Elrahim et al. 2024). Additionally, PMMA denture bases fabricated via conventional heat polymerization, CAD/CAM additive, and CAD/CAM subtractive techniques showed significant changes in surface roughness when exposed to sodium hypochlorite, particularly in the conventional and subtractive CAD/CAM groups (Takhtdar et al. 2023). These findings suggest that further investigation is warranted into the effects of commonly used cleansers such as Efferdent, Polident, and Val‐clean on surface roughness.

This investigation focused on *S. aureus* because of its increasing clinical relevance, particularly in elderly and hospitalized populations, where it has been associated with both denture stomatitis and respiratory infections. While previous studies have emphasized *Candida albicans*, this study broadens the antimicrobial scope of denture hygiene research. A study by Lewis et al. reported the presence of *S. aureus* on the dentures of 27% of outpatients and 33% of inpatients (Lewis et al. 2015). Notably, methicillin‐resistant *S. aureus* (MRSA) was detected in only 1% of outpatients compared to 12% of inpatients, highlighting a significant disparity. This suggests that hospital environments may contribute to an increased risk of MRSA colonization on dentures. Supporting this, a more recent study conducted at Hiroshima University Hospital involving patients aged 40 years and older found a significant difference in *S. aureus* isolation between individuals with and without dentures (Kawayanagi et al. 2025). These findings underscore the need for further investigation and highlight the importance of effective denture hygiene in preventing colonization by opportunistic pathogens, particularly in vulnerable patient populations.

Despite these strengths, several limitations must be acknowledged. This was an in vitro study using only a single bacterial species. In reality, oral biofilms are multispecies and subject to dynamic environmental conditions such as saliva flow, dietary influences, and host immune response. Additionally, only chemical disinfection was evaluated, without incorporating mechanical cleaning methods, which are standard in clinical practice. Future studies should examine multispecies biofilms, various cleaning durations, and the synergistic effects of combined mechanical and chemical cleaning methods. Investigating real‐world immersion times, different concentrations, and clinical patient scenarios will further validate these results.

Nevertheless, the bactericidal efficacy of Polident, Efferdent, and Val‐clean observed here highlights their potential role in preventing denture‐associated infections, particularly among vulnerable populations. Proper denture hygiene is not only essential for oral health but also for reducing systemic infection risks, including aspiration pneumonia.

## 5 Conclusion

Based on our findings, all denture cleaning solutions that we evaluated (Efferdent, Polident, and Val‐clean) revealed significant efficacy against microbial adhesion and exhibited bactericidal properties against *S. aureus* grown under laboratory conditions. Both milled PMMA and 3D printed denture base resins demonstrated similar bacterial counts, demonstrating comparable microbial adhesion. Further research is warranted to assess the effectiveness of these cleaning agents against a broader spectrum of microorganisms, including other bacterial species, fungi, and complex multispecies biofilms.

## Author Contributions

Sindi Vishaj, Donald Belles, Daher Antionio Queiroz, Maria Gonzalez, and Ransome van der Hoeven contributed to the design of the study, while Sindi Vishaj, Alissa Villhaur, Mandi Morris, and Ransome van der Hoeven were involved in the collection, analysis, and interpretation of the data. Sindi Vishaj and Ransome van der Hoeven wrote the original draft of the manuscript. Donald Belles, Daher Queiroz, and Maria Gonzalez critically revised the manuscript and provided important intellectual content. All authors approved the final version of the manuscript.

## Conflicts of Interest

The authors declare no conflicts of interest.

## Data Availability

The data that support the findings of this study are available from the corresponding author upon reasonable request.
